# Supplementary material for: Influence of solution efficiency and valence of instruction on additive and subtractive solution strategies in humans, GPT-4, and GPT-4o
Source: Commun Psychol. 2026 Jan 28;4:41. doi: 10.1038/s44271-026-00403-0 (PMC12954086; doi:10.1038/s44271-026-00403-0)
Supplement: Supplementary file 2 — Supplementary Information [file 44271_2026_403_MOESM2_ESM.pdf]

Supplementary Information (SI)

Influence of Solution Efficiency and Valence of Instruction on Additive and Subtractive Solution Strategies in Humans, GPT-4, and GPT-4o

Lydia Uhler [a, b]

Verena Jordan [a]

Jürgen Buder [c]

Markus Huff [a, c]

Frank Papenmeier [a]

[a] Department of Psychology, University of Tübingen, Schleichstraße 4, 72076 Tübingen, Germany.

[b] *Current affiliation*: Department of Psychology, University of Münster, Fliednerstraße 21, 48149 Münster, Germany.

[c] Leibniz-Institut für Wissensmedien, Schleichstraße 6, 72076 Tübingen, Germany.

Corresponding author

Lydia Uhler

Department of Psychology, University of Münster, Fliednerstraße 21, 48149 Münster, Germany.

E-Mail: lydia.uhler@uni-muenster.de

The following note applies to all tables (SI 1 and SI 3): add and sub equally eff = addition and subtraction equally efficient; sub more eff = subtraction more efficient (than addition); val neutral = valence of instruction neutral; val positive = valence of instruction positive.

The following note applies to all materials (SI 2 and SI 4): The formatting (bold and cursive fonts) is as it was presented to the human participants. To GPT-4 and GPT-4o, the material was given as input within the OpenAI API without any bold or cursive (see scripts in OSF: https://osf.io/c78rm/).

SI 1: Exclusions of human participants and GPT-4 iterations in Study 1

Table A1

Detailed Breakdown of Human Participant Exclusions in Experiment 1a, 1b, 2a, 2b

	Before exclusion	Double partici-pation	No initial consent	No final consent	Page lost focus	Equal field/word count [a]	Final sample
Experiment 1a							
add and sub equally eff	191	0	0	1	31	92	67
sub more eff	124	1	1	3	19	35	65
**Total**	**315**	**1**	**1**	**4**	**50**	**127**	**132**
Experiment 1b							
add and sub equally eff	107	0	0	0	17	3	87
sub more eff	99	0	0	1	12	2	84
**Total**	**206**	**0**	**0**	**1**	**29**	**5**	**171**
Experiment 2a							
val neutral	178	0	0	1	34	83	60
val positive	174	0	0	1	28	86	59
**Total**	**352**	**0**	**0**	**2**	**62**	**169**	**119**
Experiment 2b							
val neutral	107	0	1	0	17	6	83
val positive	121	0	0	0	32	6	83
**Total**	**228**	**0**	**1**	**0**	**49**	**12**	**166**

Note. [a] "field" in Experiment 1a and 2a (symmetry task), "word" in Experiment 1b and 2b (summary task)

Table A2

Detailed Breakdown of GPT-4 Iteration Exclusions in Experiment 1a, 1b, 2a, 2b

	Before exclusion	Incomplete answers	Equal field/word count [a]	Final sample
Experiment 1a				
add and sub equally eff	107	0	22	85
sub more eff	94	0	9	85
**Total**	201	0	31	170
Experiment 1b				
add and sub equally eff	91	0	6	85
sub more eff	86	0	1	85
**Total**	177	0	7	170
Experiment 2a				
val neutral	112	1	26	85
val positive	112	1	26	85
**Total**	222	2	52	170
Experiment 2b				
val neutral	89	0	4	85
val positive	86	0	1	85
**Total**	175	0	5	170

Note. [a] “field” in Experiment 1a and 2a (symmetry task), “word” in Experiment 1b and 2b (summary task)

SI 2: Exclusions of human participants and GPT-4o iterations in Study 2

Table A3

Detailed Breakdown of Human Participant Exclusions in Experiment 3a

	Before exclusion	Double partici-pation	No initial consent	No final consent	Bot check[a]	Page lost focus	Past first exclusion	Equal field count (text/grid)	Unequal solution of text and grid	Final sample (text/grid)
add and sub equally eff + val neutral	193	0	0	1	5	51	136	65 / 4	1	66 / 0
sub more eff + val neutral	188	0	0	3	0	49	136	39 / 2	0	93 / 2
add and sub equally eff + val positive	194	1	0	1	0+1[a]	56	135	76 / 2	0	57 / 0
sub more eff + val positive	197	0	0	3	2	54	138	44 / 2	1	87 / 4
**Total**	**772**	**1**	**0**	**8**	**7+1**[a]	**210**	**545**	**224 / 10**	**2**	**303 / 6**

Note. [a] We embedded a hidden attention-check in white font into the experiment stating that one would need to enter the wort “Strawberry” into the following text field (which was also hidden). All cases entering “Strawberry” into this text field were removed as they could potentially stem from automated bots. The “+1” refers to one case that we also removed that did not write “Strawberry” but rather “strawberry” in lowercase.

Table A4

Detailed Breakdown of Human Participant Exclusions in Experiment 3b

	Before exclusion	Double partici-pation	No initial consent	No final consent	Bot check[a]	Page lost focus	Past first exclusion	Equal word count	Final sample
add and sub equally eff + val neutral	194	4	0	2	4+2[a]	69	113	11	102
sub more eff + val neutral	198	3	0	1	1	71	122	15	107
add and sub equally eff + val positive	189	2	0	4	3	48	132	18	114
sub more eff + val positive	195	3	0	2	2+1[a]	53	134	15	119
**Total**	**776**	**12**	**0**	**9**	**10+3**[a]	**241**	**501**	**59**	442

Note. [a] We embedded a hidden attention-check in white font into the experiment stating that one would need to enter the wort “Strawberry” into the following text field (which was also hidden). All cases entering “Strawberry” into this text field were removed as they could potentially stem from automated bots. The “+” numbers refer to cases that we also removed that did not exactly write “Strawberry” but entered it in lowercase or with a spelling error.

Table A5

Detailed Breakdown of GPT-4o Iteration Exclusions in Experiment 3a

	Before exclusion	Incomplete answers	Only one response format [a]	Equal field count	Valid trials [b]	Final sample
add and sub equally eff + val neutral	160	0	1[a]	18	141	135
sub more eff + val neutral	155	0	0	8	147	135
add and sub equally eff + val positive	165	0	0	19	146	135
sub more eff + val positive	145	0	0	4	141	135
**Total**	**625**	**0**	**1**	**49**	**575**	**540**

Note. [a] In one trial, GPT-4o output contained only a text-based solution, no grid-based solution. [b] The raters coded the first 135 trials, then in a second round, the number of excluded trials and additional trials were collected according to the exclusion rate. For this reason, the number of valid trials is higher than the final sample. From "Valid Trials" to "Final Sample," the lower trials were truncated after the 135th trial.

Table A6

Detailed Breakdown of GPT-4o Iteration Exclusions in Experiment 3b

	Before exclusion	Incomplete answers	Equal word count	Final sample
add and sub equally eff + val neutral	137	0	2	135
sub more eff + val neutral	137	0	2	135
add and sub equally eff + val positive	136	0	1	135
sub more eff + val positive	135	0	0	135
**Total**	**545**	**0**	**5**	**540**

SI 3: Material Study 1

Symmetry Task – Variation of Solution Efficiency (Experiment 1a)

Condition: Addition and Subtraction Equally Efficient

There is a digital 4-by-4 grid with columns labeled A through D from left to right and rows labeled 1 through 4 from top to bottom. You can switch any field from “[X]” to “[ ]” or from “[ ]” to “[X]“. Below you can see the current pattern of fields.

A B C D

1 [X] [ ] [ ] [ ]

2 [ ] [X] [X] [ ]

3 [ ] [X] [X] [ ]

4 [X] [ ] [ ] [ ]

Your task is to <u>change</u> the pattern of the fields so that the grid is perfectly symmetrical from left to right and from top to bottom, that is, it must be exactly mirror symmetrical both horizontally and vertically. There are many technically correct solutions to this puzzle. However, your task is to create symmetry with as little switching as possible.

Please describe how you solve the task in the following text box.

[For human participants, text box was provided]

[For GPT-4] Text box:

Condition: Subtraction More Efficient

There is a digital 4-by-4 grid with columns labeled A through D from left to right and rows labeled 1 through 4 from top to bottom. You can switch any field from “[X]” to “[ ]” or from “[ ]” to “[X]“. Below you can see the current pattern of fields.

```
   A   B   C   D
1 [X] [ ] [ ] [ ]
2 [ ] [X] [X] [ ]
3 [ ] [X] [X] [ ]
4 [ ] [ ] [ ] [ ]
```

Your task is to <u>change</u> the pattern of the fields so that the grid is perfectly symmetrical from left to right and from top to bottom, that is, it must be exactly mirror symmetrical both horizontally and vertically. There are many technically correct solutions to this puzzle. However, your task is to create symmetry with as little switching as possible.

Please describe how you solve the task in the following text box.

[For human participants, text box was provided]

[For GPT-4] Text box:

Symmetry Task – Variation of Valence of Instruction (Experiment 2a)

Condition: Valence Neutral

There is a digital 4-by-4 grid with columns labeled A through D from left to right and rows labeled 1 through 4 from top to bottom. You can switch any field from “[X]” to “[ ]” or from “[ ]” to “[X]“. Below you can see the current pattern of fields.

```
    A   B   C   D
1  [X] [ ] [ ] [ ]
2  [ ] [X] [X] [ ]
3  [ ] [X] [X] [ ]
4  [X] [ ] [ ] [ ]
```

Your task is to change the pattern of the fields so that the grid is perfectly symmetrical from left to right and from top to bottom, that is, it must be exactly mirror symmetrical both horizontally and vertically. There are many technically correct solutions to this puzzle. However, your task is to create symmetry with as little switching as possible.

Please describe how you solve the task in the following text box.

[For human participants, text box was provided]

[For GPT-4] Text box:

Condition: Valence Positive

There is a digital 4-by-4 grid with columns labeled A through D from left to right and rows labeled 1 through 4 from top to bottom. You can switch any field from "[X]" to "[ ]" or from "[ ]" to "[X]". Below you can see the current pattern of fields.

A B C D

1 [X] [ ] [ ] [ ]

2 [ ] [X] [X] [ ]

3 [ ] [X] [X] [ ]

4 [X] [ ] [ ] [ ]

Your task is to <u>improve</u> the pattern of the fields so that the grid is perfectly symmetrical from left to right and from top to bottom, that is, it must be exactly mirror symmetrical both horizontally and vertically. There are many technically correct solutions to this puzzle. However, your task is to create symmetry with as little switching as possible.

Please describe how you solve the task in the following text box.

[For human participants, text box was provided]

[For GPT-4] Text box:

Summary Task – Variation of Solution Efficiency (Experiment 1b)

Condition: Addition and Subtraction Equally Efficient

Here is a short news article about fire fighting in New South Wales, Australia. <u>Please read it carefully.</u>

[For human participants, this sentence was included: "**Note: The 'Next' button appears in 60 seconds.**"]

'Thor' aircraft, capable of dropping 15,000 litres of water, arrives in NSW ahead of bushfire season

(By Nick Dole, ABC News, Sep 1, 2015)

The New South Wales Rural Fire Service has unveiled its newest firefighting tool, a massive water-bombing aircraft nicknamed Thor, ahead of the bushfire season. The plane has just arrived from North America for the Australian bushfire season and performed a practise water-bombing exercise near Richmond air base in north-west Sydney. The air tanker can drop more than 15,000 litres of water or fire retardant on a blaze within a few seconds. It has a loaded cruising speed of 545 kilometres an hour, meaning it will be able to reach most fires in the state within one hour. Emergency Services Minister David Elliott said the aircraft would be a welcome sight for residents in fire-prone areas. "The people of NSW will be able to rest easy tonight, the first day of spring, knowing that Thor will be in the skies looking out for them," Mr Elliott said. RFS Commissioner Shane Fitzsimmons said the specially configured C-130 Hercules will dwarf the capability of the smaller water-bombing planes in the fleet. "This can carry five times the water of those machines," he said. "They can take off and land on airstrips of less than two kilometres, they're very versatile." The plane's pilot, Rickey Rau said Thor can fly fully loaded for at least five hours. "Depending on where the fire is, we can launch out of here fully loaded and do two to three runs on the fire before needing fuel," Mr Rau said. The plane will be based at the Richmond air base, but could also be moved to smaller bases at Williamtown, Tamworth, Dubbo and Canberra. The aircraft will be leased to NSW for two fire seasons as part of a $10 million State Government funding package. An ever bigger DC-10 air tanker will arrive in October. Commissioner Fitzsimmons said much of NSW was facing an "above normal" risk of bushfires this season. "That's

largely caused by the moisture deficit," he said. He said there has been solid rainfall in recent months, but it was not likely to have a lasting effect. "With the onset of a strengthening El Nino still dominating the forecast for this season, the moisture is expected to be depleted fairly quickly, giving rise to a difficult fire season for 2015/16," he said.

[Next page for human participants]

This is again the article you have just read. Below the article you will find a summary of the article.

'Thor' aircraft, capable of dropping 15,000 litres of water, arrives in NSW ahead of bushfire season

(By Nick Dole, ABC News, Sep 1, 2015)

The New South Wales Rural Fire Service has unveiled its newest firefighting tool, a massive water-bombing aircraft nicknamed Thor, ahead of the bushfire season. The plane has just arrived from North America for the Australian bushfire season and performed a practise water-bombing exercise near Richmond air base in north-west Sydney. The air tanker can drop more than 15,000 litres of water or fire retardant on a blaze within a few seconds. It has a loaded cruising speed of 545 kilometres an hour, meaning it will be able to reach most fires in the state within one hour. Emergency Services Minister David Elliott said the aircraft would be a welcome sight for residents in fire-prone areas. "The people of NSW will be able to rest easy tonight, the first day of spring, knowing that Thor will be in the skies looking out for them," Mr Elliott said. RFS Commissioner Shane Fitzsimmons said the specially configured C-130 Hercules will dwarf the capability of the smaller water-bombing planes in the fleet. "This can carry five times the water of those machines," he said. "They can take off and land on airstrips of less than two kilometres, they're very versatile." The plane's pilot, Rickey Rau said Thor can fly fully loaded for at least five hours. "Depending on where the fire is, we can launch out of here fully loaded and do two to three runs on the fire before needing fuel," Mr Rau said. The plane will be based at the Richmond air base, but could also be moved to smaller bases at Williamtown, Tamworth, Dubbo and Canberra. The aircraft will be leased to NSW for two fire seasons as part of a $10 million State Government funding package. An ever bigger DC-10 air tanker will arrive in October. Commissioner Fitzsimmons said much of NSW was facing an "above normal" risk of bushfires this season. "That's largely caused by the moisture deficit," he said. He said there has been solid rainfall in recent months, but it was not likely to have a lasting effect. "With the onset of a strengthening El Nino still dominating

the forecast for this season, the moisture is expected to be depleted fairly quickly, giving rise to a difficult fire season for 2015/16," he said.

SUMMARY:

According to ABC's Nick Dole, September 1st, the New South Wales Fire Service will use the first of two fire-fighting US-made air tankers this spring. Lack of moisture and El Nino signal more risk of fire. But the plane, reaching any fire in NSW quickly and dumping thousands of litres quickly, will make people feel safer. Fuel efficiency, range, speed, loading capacity and its ability to use short airstrips make it a singularly versatile fire-fighting tool.

YOUR TASK: We would like you to edit this summary. For your convenience, we have pasted it into the box below.

NOTE: The summary currently contains 76 words. The edited summary MUST NOT be between 64 and 88 words long.

[For human participants, text box with editable summary was provided]

[For GPT-4] Text box:

According to ABC's Nick Dole, September 1st, the New South Wales Fire Service will use the first of two fire-fighting US-made air tankers this spring. Lack of moisture and El Nino signal more risk of fire. But the plane, reaching any fire in NSW quickly and dumping thousands of litres quickly, will make people feel safer. Fuel efficiency, range, speed, loading capacity and its ability to use short airstrips make it a singularly versatile fire-fighting tool.

Condition: Subtraction More Efficient

Here is a short news article about fire fighting in New South Wales, Australia. <u>Please read it carefully.</u>

[For human participants, this sentence was included: "**Note: The 'Next' button appears in 60 seconds.**"]

'Thor' aircraft, capable of dropping 15,000 litres of water, arrives in NSW ahead of bushfire season

(By Nick Dole, ABC News, Sep 1, 2015)

The New South Wales Rural Fire Service has unveiled its newest firefighting tool, a massive water-bombing aircraft nicknamed Thor, ahead of the bushfire season. The plane has just arrived from North America for the Australian bushfire season and performed a practise water-bombing exercise near Richmond air base in north-west Sydney. The air tanker can drop more than 15,000 litres of water or fire retardant on a blaze within a few seconds. It has a loaded cruising speed of 545 kilometres an hour, meaning it will be able to reach most fires in the state within one hour. Emergency Services Minister David Elliott said the aircraft would be a welcome sight for residents in fire-prone areas. "The people of NSW will be able to rest easy tonight, the first day of spring, knowing that Thor will be in the skies looking out for them," Mr Elliott said. RFS Commissioner Shane Fitzsimmons said the specially configured C-130 Hercules will dwarf the capability of the smaller water-bombing planes in the fleet. "This can carry five times the water of those machines," he said. "They can take off and land on airstrips of less than two kilometres, they're very versatile." The plane's pilot, Rickey Rau said Thor can fly fully loaded for at least five hours. "Depending on where the fire is, we can launch out of here fully loaded and do two to three runs on the fire before needing fuel," Mr Rau said. The plane will be based at the Richmond air base, but could also be moved to smaller bases at Williamtown, Tamworth, Dubbo and Canberra. The aircraft will be leased to NSW for two fire seasons as part of a $10 million State Government funding package. An ever bigger DC-10 air tanker will arrive in October. Commissioner Fitzsimmons said much of NSW was facing an "above normal" risk of bushfires this season. "That's largely caused by the moisture deficit," he said. He said there has been solid rainfall in recent months, but it was not likely to have a lasting effect. "With the onset of a strengthening El Nino still dominating

the forecast for this season, the moisture is expected to be depleted fairly quickly, giving rise to a difficult fire season for 2015/16," he said.

[Next page for human participants]

This is again the article you have just read. Below the article you will find a summary of the article.

'Thor' aircraft, capable of dropping 15,000 litres of water, arrives in NSW ahead of bushfire season

(By Nick Dole, ABC News, Sep 1, 2015)

The New South Wales Rural Fire Service has unveiled its newest firefighting tool, a massive water-bombing aircraft nicknamed Thor, ahead of the bushfire season. The plane has just arrived from North America for the Australian bushfire season and performed a practise water-bombing exercise near Richmond air base in north-west Sydney. The air tanker can drop more than 15,000 litres of water or fire retardant on a blaze within a few seconds. It has a loaded cruising speed of 545 kilometres an hour, meaning it will be able to reach most fires in the state within one hour. Emergency Services Minister David Elliott said the aircraft would be a welcome sight for residents in fire-prone areas. "The people of NSW will be able to rest easy tonight, the first day of spring, knowing that Thor will be in the skies looking out for them," Mr Elliott said. RFS Commissioner Shane Fitzsimmons said the specially configured C-130 Hercules will dwarf the capability of the smaller water-bombing planes in the fleet. "This can carry five times the water of those machines," he said. "They can take off and land on airstrips of less than two kilometres, they're very versatile." The plane's pilot, Rickey Rau said Thor can fly fully loaded for at least five hours. "Depending on where the fire is, we can launch out of here fully loaded and do two to three runs on the fire before needing fuel," Mr Rau said. The plane will be based at the Richmond air base, but could also be moved to smaller bases at Williamtown, Tamworth, Dubbo and Canberra. The aircraft will be leased to NSW for two fire seasons as part of a $10 million State Government funding package. An ever bigger DC-10 air tanker will arrive in October. Commissioner Fitzsimmons said much of NSW was facing an "above normal" risk of bushfires this season. "That's largely caused by the moisture deficit," he said. He said there has been solid rainfall in recent months, but it was not likely to have a lasting effect. "With the onset of a strengthening El Nino still dominating the forecast for this season, the moisture is expected to be depleted fairly quickly, giving rise to a difficult fire season for 2015/16," he said.

SUMMARY:

According to ABC’s Nick Dole, September 1st, the New South Wales Fire Service will use the first of two fire-fighting US-made air tankers this spring. Lack of moisture and El Nino signal more risk of fire. But the plane, reaching any fire in NSW quickly and dumping thousands of litres quickly, will make people feel safer. Fuel efficiency, range, speed, loading capacity and its ability to use short airstrips make it a singularly versatile fire-fighting tool.

YOUR TASK: We would like you to edit this summary. For your convenience, we have pasted it into the box below.

NOTE: The summary currently contains 76 words. The edited summary MUST NOT be between 70 and 94 words long.

[For human participants, text box with editable summary was provided]

[For GPT-4] Text box:

According to ABC’s Nick Dole, September 1st, the New South Wales Fire Service will use the first of two fire-fighting US-made air tankers this spring. Lack of moisture and El Nino signal more risk of fire. But the plane, reaching any fire in NSW quickly and dumping thousands of litres quickly, will make people feel safer. Fuel efficiency, range, speed, loading capacity and its ability to use short airstrips make it a singularly versatile fire-fighting tool.

Summary Task – Variation of Valence of Instruction (Experiment 2b)

Condition: Valence Neutral

Here is a short news article about fire fighting in New South Wales, Australia. <u>Please read it carefully.</u>

[For human participants, this sentence was included: "**Note: The 'Next' button appears in 60 seconds.**"]

'Thor' aircraft, capable of dropping 15,000 litres of water, arrives in NSW ahead of bushfire season

(By Nick Dole, ABC News, Sep 1, 2015)

The New South Wales Rural Fire Service has unveiled its newest firefighting tool, a massive water-bombing aircraft nicknamed Thor, ahead of the bushfire season. The plane has just arrived from North America for the Australian bushfire season and performed a practise water-bombing exercise near Richmond air base in north-west Sydney. The air tanker can drop more than 15,000 litres of water or fire retardant on a blaze within a few seconds. It has a loaded cruising speed of 545 kilometres an hour, meaning it will be able to reach most fires in the state within one hour. Emergency Services Minister David Elliott said the aircraft would be a welcome sight for residents in fire-prone areas. "The people of NSW will be able to rest easy tonight, the first day of spring, knowing that Thor will be in the skies looking out for them," Mr Elliott said. RFS Commissioner Shane Fitzsimmons said the specially configured C-130 Hercules will dwarf the capability of the smaller water-bombing planes in the fleet. "This can carry five times the water of those machines," he said. "They can take off and land on airstrips of less than two kilometres, they're very versatile." The plane's pilot, Rickey Rau said Thor can fly fully loaded for at least five hours. "Depending on where the fire is, we can launch out of here fully loaded and do two to three runs on the fire before needing fuel," Mr Rau said. The plane will be based at the Richmond air base, but could also be moved to smaller bases at Williamtown, Tamworth, Dubbo and Canberra. The aircraft will be leased to NSW for two fire seasons as part of a $10 million State Government funding package. An ever bigger DC-10 air tanker will arrive in October. Commissioner Fitzsimmons said much of NSW was facing an "above normal" risk of bushfires this season. "That's

largely caused by the moisture deficit," he said. He said there has been solid rainfall in recent months, but it was not likely to have a lasting effect. "With the onset of a strengthening El Nino still dominating the forecast for this season, the moisture is expected to be depleted fairly quickly, giving rise to a difficult fire season for 2015/16," he said.

[Next page for human participants]

This is again the article you have just read. Below the article you will find a summary of the article.

'Thor' aircraft, capable of dropping 15,000 litres of water, arrives in NSW ahead of bushfire season

(By Nick Dole, ABC News, Sep 1, 2015)

The New South Wales Rural Fire Service has unveiled its newest firefighting tool, a massive water-bombing aircraft nicknamed Thor, ahead of the bushfire season. The plane has just arrived from North America for the Australian bushfire season and performed a practise water-bombing exercise near Richmond air base in north-west Sydney. The air tanker can drop more than 15,000 litres of water or fire retardant on a blaze within a few seconds. It has a loaded cruising speed of 545 kilometres an hour, meaning it will be able to reach most fires in the state within one hour. Emergency Services Minister David Elliott said the aircraft would be a welcome sight for residents in fire-prone areas. “The people of NSW will be able to rest easy tonight, the first day of spring, knowing that Thor will be in the skies looking out for them,” Mr Elliott said. RFS Commissioner Shane Fitzsimmons said the specially configured C-130 Hercules will dwarf the capability of the smaller water-bombing planes in the fleet. “This can carry five times the water of those machines,” he said. “They can take off and land on airstrips of less than two kilometres, they’re very versatile.” The plane’s pilot, Rickey Rau said Thor can fly fully loaded for at least five hours. “Depending on where the fire is, we can launch out of here fully loaded and do two to three runs on the fire before needing fuel,” Mr Rau said. The plane will be based at the Richmond air base, but could also be moved to smaller bases at Williamtown, Tamworth, Dubbo and Canberra. The aircraft will be leased to NSW for two fire seasons as part of a $10 million State Government funding package. An ever bigger DC-10 air tanker will arrive in October. Commissioner Fitzsimmons said much of NSW was facing an “above normal” risk of bushfires this season. “That’s largely caused by the moisture deficit,” he said. He said there has been solid rainfall in recent months, but it was not likely to have a lasting effect. “With the onset of a strengthening El Nino still dominating

the forecast for this season, the moisture is expected to be depleted fairly quickly, giving rise to a difficult fire season for 2015/16," he said.

SUMMARY:

According to ABC's Nick Dole, September 1st, the New South Wales Fire Service will use the first of two fire-fighting US-made air tankers this spring. Lack of moisture and El Nino signal more risk of fire. But the plane, reaching any fire in NSW quickly and dumping thousands of litres quickly, will make people feel safer. Fuel efficiency, range, speed, loading capacity and its ability to use short airstrips make it a singularly versatile fire-fighting tool.

YOUR TASK: We would like you to edit this summary. For your convenience, we have pasted it into the box below.

NOTE: The summary currently contains 76 words. The edited summary MUST NOT be between 64 and 88 words long.

[For human participants, text box with editable summary was provided]

[For GPT-4] Text box:

According to ABC's Nick Dole, September 1st, the New South Wales Fire Service will use the first of two fire-fighting US-made air tankers this spring. Lack of moisture and El Nino signal more risk of fire. But the plane, reaching any fire in NSW quickly and dumping thousands of litres quickly, will make people feel safer. Fuel efficiency, range, speed, loading capacity and its ability to use short airstrips make it a singularly versatile fire-fighting tool.

Condition: Valence Positive

Here is a short news article about fire fighting in New South Wales, Australia. <u>Please read it carefully.</u>

[For human participants, this sentence was included: “**Note: The 'Next' button appears in 60 seconds.“**]

'Thor' aircraft, capable of dropping 15,000 litres of water, arrives in NSW ahead of bushfire season

(By Nick Dole, ABC News, Sep 1, 2015)

The New South Wales Rural Fire Service has unveiled its newest firefighting tool, a massive water-bombing aircraft nicknamed Thor, ahead of the bushfire season. The plane has just arrived from North America for the Australian bushfire season and performed a practise water-bombing exercise near Richmond air base in north-west Sydney. The air tanker can drop more than 15,000 litres of water or fire retardant on a blaze within a few seconds. It has a loaded cruising speed of 545 kilometres an hour, meaning it will be able to reach most fires in the state within one hour. Emergency Services Minister David Elliott said the aircraft would be a welcome sight for residents in fire-prone areas. "The people of NSW will be able to rest easy tonight, the first day of spring, knowing that Thor will be in the skies looking out for them," Mr Elliott said. RFS Commissioner Shane Fitzsimmons said the specially configured C-130 Hercules will dwarf the capability of the smaller water-bombing planes in the fleet. "This can carry five times the water of those machines," he said. "They can take off and land on airstrips of less than two kilometres, they're very versatile." The plane's pilot, Rickey Rau said Thor can fly fully loaded for at least five hours. "Depending on where the fire is, we can launch out of here fully loaded and do two to three runs on the fire before needing fuel," Mr Rau said. The plane will be based at the Richmond air base, but could also be moved to smaller bases at Williamtown, Tamworth, Dubbo and Canberra. The aircraft will be leased to NSW for two fire seasons as part of a $10 million State Government funding package. An ever bigger DC-10 air tanker will arrive in October. Commissioner Fitzsimmons said much of NSW was facing an "above normal" risk of bushfires this season. "That's largely caused by the moisture deficit," he said. He said there has been solid rainfall in recent months, but it was not likely to have a lasting effect. "With the onset of a strengthening El Nino still dominating

the forecast for this season, the moisture is expected to be depleted fairly quickly, giving rise to a difficult fire season for 2015/16," he said.

[Next page for human participants]

This is again the article you have just read. Below the article you will find a summary of the article.

'Thor' aircraft, capable of dropping 15,000 litres of water, arrives in NSW ahead of bushfire season

(By Nick Dole, ABC News, Sep 1, 2015)

The New South Wales Rural Fire Service has unveiled its newest firefighting tool, a massive water-bombing aircraft nicknamed Thor, ahead of the bushfire season. The plane has just arrived from North America for the Australian bushfire season and performed a practise water-bombing exercise near Richmond air base in north-west Sydney. The air tanker can drop more than 15,000 litres of water or fire retardant on a blaze within a few seconds. It has a loaded cruising speed of 545 kilometres an hour, meaning it will be able to reach most fires in the state within one hour. Emergency Services Minister David Elliott said the aircraft would be a welcome sight for residents in fire-prone areas. "The people of NSW will be able to rest easy tonight, the first day of spring, knowing that Thor will be in the skies looking out for them," Mr Elliott said. RFS Commissioner Shane Fitzsimmons said the specially configured C-130 Hercules will dwarf the capability of the smaller water-bombing planes in the fleet. "This can carry five times the water of those machines," he said. "They can take off and land on airstrips of less than two kilometres, they're very versatile." The plane's pilot, Rickey Rau said Thor can fly fully loaded for at least five hours. "Depending on where the fire is, we can launch out of here fully loaded and do two to three runs on the fire before needing fuel," Mr Rau said. The plane will be based at the Richmond air base, but could also be moved to smaller bases at Williamtown, Tamworth, Dubbo and Canberra. The aircraft will be leased to NSW for two fire seasons as part of a $10 million State Government funding package. An ever bigger DC-10 air tanker will arrive in October. Commissioner Fitzsimmons said much of NSW was facing an "above normal" risk of bushfires this season. "That's largely caused by the moisture deficit," he said. He said there has been solid rainfall in recent months, but it was not likely to have a lasting effect. "With the onset of a strengthening El Nino still dominating the forecast for this season, the moisture is expected to be depleted fairly quickly, giving rise to a difficult fire season for 2015/16," he said.

SUMMARY:

According to ABC’s Nick Dole, September 1st, the New South Wales Fire Service will use the first of two fire-fighting US-made air tankers this spring. Lack of moisture and El Nino signal more risk of fire. But the plane, reaching any fire in NSW quickly and dumping thousands of litres quickly, will make people feel safer. Fuel efficiency, range, speed, loading capacity and its ability to use short airstrips make it a singularly versatile fire-fighting tool.

YOUR TASK: We would like you to improve this summary. For your convenience, we have pasted it into the box below.

NOTE: The summary currently contains 76 words. The improved summary MUST NOT be between 64 and 88 words long.

[For human participants, text box with editable summary was provided]

[For GPT-4] Text box:

According to ABC’s Nick Dole, September 1st, the New South Wales Fire Service will use the first of two fire-fighting US-made air tankers this spring. Lack of moisture and El Nino signal more risk of fire. But the plane, reaching any fire in NSW quickly and dumping thousands of litres quickly, will make people feel safer. Fuel efficiency, range, speed, loading capacity and its ability to use short airstrips make it a singularly versatile fire-fighting tool.

SI 4: Material Study 2

Symmetry Task – Variation of Solution Efficiency and Valence of Instruction (Experiment 3a)

Condition: Addition and Subtraction Equally Efficient + Valence of Instruction Neutral

There is a digital 4-by-4 grid with columns labeled A through D from left to right and rows labeled 1 through 4 from top to bottom. Below you can see the current pattern of fields.

A B C D

1 [X] [ ] [ ] [ ]

2 [ ] [X] [X] [ ]

3 [ ] [X] [X] [ ]

4 [X] [ ] [ ] [ ]

You can toggle any field from "[X]" to "[ ]" or from "[ ]" to "[X]" (i.e., you cannot move a "[X]" or a "[ ]", only toggle the fields).

Your task is to <u>change</u> the pattern of the fields so that the grid is perfectly symmetrical from left to right and from top to bottom, that is, it must be exactly mirror symmetrical both horizontally and vertically. There are many technically correct solutions to change the pattern. However, your task is to create symmetry with as few changes as possible.

Please describe how you change the pattern in the following text box.

[For human participants, text box was provided]

[For GPT-4o] Text box:

Condition: Subtraction More Efficient + Valence of Instruction Neutral

There is a digital 4-by-4 grid with columns labeled A through D from left to right and rows labeled 1 through 4 from top to bottom. Below you can see the current pattern of fields.

	A	B	C	D
1	[X]	[ ]	[ ]	[ ]
2	[ ]	[X]	[X]	[ ]
3	[ ]	[X]	[X]	[ ]
4	[ ]	[ ]	[ ]	[ ]

You can toggle any field from “[X]” to “[ ]” or from “[ ]” to “[X]” (i.e., you cannot move a “[X]” or a “[ ]“, only toggle the fields).

Your task is to <u>change</u> the pattern of the fields so that the grid is perfectly symmetrical from left to right and from top to bottom, that is, it must be exactly mirror symmetrical both horizontally and vertically. There are many technically correct solutions to change the pattern. However, your task is to create symmetry with as few changes as possible.

Please describe how you change the pattern in the following text box.

[For human participants, text box was provided]

[For GPT-4o] Text box:

Condition: Addition and Subtraction Equally Efficient + Valence of Instruction Positive

There is a digital 4-by-4 grid with columns labeled A through D from left to right and rows labeled 1 through 4 from top to bottom. Below you can see the current pattern of fields.

A B C D

1 [X] [ ] [ ] [ ]

2 [ ] [X] [X] [ ]

3 [ ] [X] [X] [ ]

4 [X] [ ] [ ] [ ]

You can toggle any field from "[X]" to "[ ]" or from "[ ]" to "[X]" (i.e., you cannot move a "[X]" or a "[ ]", only toggle the fields).

Your task is to <u>improve</u> the pattern of the fields so that the grid is perfectly symmetrical from left to right and from top to bottom, that is, it must be exactly mirror symmetrical both horizontally and vertically. There are many technically correct solutions to improve the pattern. However, your task is to create symmetry with as few improvements as possible.

Please describe how you improve the pattern in the following text box.

[For human participants, text box was provided]

[For GPT-4o] Text box:

Condition: Subtraction More Efficient + Valence of Instruction Positive

There is a digital 4-by-4 grid with columns labeled A through D from left to right and rows labeled 1 through 4 from top to bottom. Below you can see the current pattern of fields.

A B C D

1 [X] [ ] [ ] [ ]

2 [ ] [X] [X] [ ]

3 [ ] [X] [X] [ ]

4 [ ] [ ] [ ] [ ]

You can toggle any field from “[X]” to “[ ]” or from “[ ]” to “[X]” (i.e., you cannot move a “[X]” or a “[ ]“, only toggle the fields).

Your task is to <u>improve</u> the pattern of the fields so that the grid is perfectly symmetrical from left to right and from top to bottom, that is, it must be exactly mirror symmetrical both horizontally and vertically. There are many technically correct solutions to improve the pattern. However, your task is to create symmetry with as few improvements as possible.

Please describe how you improve the pattern in the following text box.

[For human participants, text box was provided]

[For GPT-4o] Text box:

Summary Task – Variation of Solution Efficiency and Valence of Instruction (Experiment 3b)

Condition: Addition and Subtraction Equally Efficient + Valence of Instruction Neutral

Here is a short news article about fire fighting in New South Wales, Australia. <u>Please read it carefully.</u>

[For human participants, this sentence was included: “**Note: The 'Next' button appears in 60 seconds.“**]

'Thor' aircraft, capable of dropping 15,000 litres of water, arrives in NSW ahead of bushfire season

(By Nick Dole, ABC News, Sep 1, 2015)

The New South Wales Rural Fire Service has unveiled its newest firefighting tool, a massive water-bombing aircraft nicknamed Thor, ahead of the bushfire season. The plane has just arrived from North America for the Australian bushfire season and performed a practise water-bombing exercise near Richmond air base in north-west Sydney. The air tanker can drop more than 15,000 litres of water or fire retardant on a blaze within a few seconds. It has a loaded cruising speed of 545 kilometres an hour, meaning it will be able to reach most fires in the state within one hour. Emergency Services Minister David Elliott said the aircraft would be a welcome sight for residents in fire-prone areas. "The people of NSW will be able to rest easy tonight, the first day of spring, knowing that Thor will be in the skies looking out for them," Mr Elliott said. RFS Commissioner Shane Fitzsimmons said the specially configured C-130 Hercules will dwarf the capability of the smaller water-bombing planes in the fleet. "This can carry five times the water of those machines," he said. "They can take off and land on airstrips of less than two kilometres, they're very versatile." The plane's pilot, Rickey Rau said Thor can fly fully loaded for at least five hours. "Depending on where the fire is, we can launch out of here fully loaded and do two to three runs on the fire before needing fuel," Mr Rau said. The plane will be based at the Richmond air base, but could also be moved to smaller bases at Williamtown, Tamworth, Dubbo and Canberra. The aircraft will be leased to NSW for two fire seasons as part of a $10 million State Government funding package. An ever bigger DC-10 air tanker will arrive in October. Commissioner Fitzsimmons said much of NSW was facing an "above normal" risk of bushfires this season. "That's

largely caused by the moisture deficit," he said. He said there has been solid rainfall in recent months, but it was not likely to have a lasting effect. "With the onset of a strengthening El Nino still dominating the forecast for this season, the moisture is expected to be depleted fairly quickly, giving rise to a difficult fire season for 2015/16," he said.

[Next page for human participants]

This is again the article you have just read. Below the article you will find a summary of the article.

'Thor' aircraft, capable of dropping 15,000 litres of water, arrives in NSW ahead of bushfire season

(By Nick Dole, ABC News, Sep 1, 2015)

The New South Wales Rural Fire Service has unveiled its newest firefighting tool, a massive water-bombing aircraft nicknamed Thor, ahead of the bushfire season. The plane has just arrived from North America for the Australian bushfire season and performed a practise water-bombing exercise near Richmond air base in north-west Sydney. The air tanker can drop more than 15,000 litres of water or fire retardant on a blaze within a few seconds. It has a loaded cruising speed of 545 kilometres an hour, meaning it will be able to reach most fires in the state within one hour. Emergency Services Minister David Elliott said the aircraft would be a welcome sight for residents in fire-prone areas. "The people of NSW will be able to rest easy tonight, the first day of spring, knowing that Thor will be in the skies looking out for them," Mr Elliott said. RFS Commissioner Shane Fitzsimmons said the specially configured C-130 Hercules will dwarf the capability of the smaller water-bombing planes in the fleet. "This can carry five times the water of those machines," he said. "They can take off and land on airstrips of less than two kilometres, they're very versatile." The plane's pilot, Rickey Rau said Thor can fly fully loaded for at least five hours. "Depending on where the fire is, we can launch out of here fully loaded and do two to three runs on the fire before needing fuel," Mr Rau said. The plane will be based at the Richmond air base, but could also be moved to smaller bases at Williamtown, Tamworth, Dubbo and Canberra. The aircraft will be leased to NSW for two fire seasons as part of a $10 million State Government funding package. An ever bigger DC-10 air tanker will arrive in October. Commissioner Fitzsimmons said much of NSW was facing an "above normal" risk of bushfires this season. "That's largely caused by the moisture deficit," he said. He said there has been solid rainfall in recent months, but it was not likely to have a lasting effect. "With the onset of a strengthening El Nino still dominating

the forecast for this season, the moisture is expected to be depleted fairly quickly, giving rise to a difficult fire season for 2015/16," he said.

SUMMARY:

According to ABC's Nick Dole, September 1st, the New South Wales Fire Service will use the first of two fire-fighting US-made air tankers this spring. Lack of moisture and El Nino signal more risk of fire. But the plane, reaching any fire in NSW quickly and dumping thousands of litres quickly, will make people feel safer. Fuel efficiency, range, speed, loading capacity and its ability to use short airstrips make it a singularly versatile fire-fighting tool.

YOUR TASK: We would like you to <u>change</u> this summary. To make it easier for you to make changes, we have pasted it in the box below so you can change it.

NOTE: The summary currently contains 76 words. The changed summary <u>MUST NOT</u> be between 64 and 88 words long.

[For human participants, text box with editable summary was provided]

[For GPT-4o] Text box:

According to ABC's Nick Dole, September 1st, the New South Wales Fire Service will use the first of two fire-fighting US-made air tankers this spring. Lack of moisture and El Nino signal more risk of fire. But the plane, reaching any fire in NSW quickly and dumping thousands of litres quickly, will make people feel safer. Fuel efficiency, range, speed, loading capacity and its ability to use short airstrips make it a singularly versatile fire-fighting tool.

Condition: Subtraction More Efficient + Valence of Instruction Neutral

Here is a short news article about fire fighting in New South Wales, Australia. <u>Please read it carefully.</u>

[For human participants, this sentence was included: "**Note: The 'Next' button appears in 60 seconds.**"]

'Thor' aircraft, capable of dropping 15,000 litres of water, arrives in NSW ahead of bushfire season

(By Nick Dole, ABC News, Sep 1, 2015)

The New South Wales Rural Fire Service has unveiled its newest firefighting tool, a massive water-bombing aircraft nicknamed Thor, ahead of the bushfire season. The plane has just arrived from North America for the Australian bushfire season and performed a practise water-bombing exercise near Richmond air base in north-west Sydney. The air tanker can drop more than 15,000 litres of water or fire retardant on a blaze within a few seconds. It has a loaded cruising speed of 545 kilometres an hour, meaning it will be able to reach most fires in the state within one hour. Emergency Services Minister David Elliott said the aircraft would be a welcome sight for residents in fire-prone areas. "The people of NSW will be able to rest easy tonight, the first day of spring, knowing that Thor will be in the skies looking out for them," Mr Elliott said. RFS Commissioner Shane Fitzsimmons said the specially configured C-130 Hercules will dwarf the capability of the smaller water-bombing planes in the fleet. "This can carry five times the water of those machines," he said. "They can take off and land on airstrips of less than two kilometres, they're very versatile." The plane's pilot, Rickey Rau said Thor can fly fully loaded for at least five hours. "Depending on where the fire is, we can launch out of here fully loaded and do two to three runs on the fire before needing fuel," Mr Rau said. The plane will be based at the Richmond air base, but could also be moved to smaller bases at Williamtown, Tamworth, Dubbo and Canberra. The aircraft will be leased to NSW for two fire seasons as part of a $10 million State Government funding package. An ever bigger DC-10 air tanker will arrive in October. Commissioner Fitzsimmons said much of NSW was facing an "above normal" risk of bushfires this season. "That's largely caused by the moisture deficit," he said. He said there has been solid rainfall in recent months, but it was not likely to have a lasting effect. "With the onset of a strengthening El Nino still dominating

the forecast for this season, the moisture is expected to be depleted fairly quickly, giving rise to a difficult fire season for 2015/16," he said.

[Next page for human participants]

This is again the article you have just read. Below the article you will find a summary of the article.

'Thor' aircraft, capable of dropping 15,000 litres of water, arrives in NSW ahead of bushfire season

(By Nick Dole, ABC News, Sep 1, 2015)

The New South Wales Rural Fire Service has unveiled its newest firefighting tool, a massive water-bombing aircraft nicknamed Thor, ahead of the bushfire season. The plane has just arrived from North America for the Australian bushfire season and performed a practise water-bombing exercise near Richmond air base in north-west Sydney. The air tanker can drop more than 15,000 litres of water or fire retardant on a blaze within a few seconds. It has a loaded cruising speed of 545 kilometres an hour, meaning it will be able to reach most fires in the state within one hour. Emergency Services Minister David Elliott said the aircraft would be a welcome sight for residents in fire-prone areas. "The people of NSW will be able to rest easy tonight, the first day of spring, knowing that Thor will be in the skies looking out for them," Mr Elliott said. RFS Commissioner Shane Fitzsimmons said the specially configured C-130 Hercules will dwarf the capability of the smaller water-bombing planes in the fleet. "This can carry five times the water of those machines," he said. "They can take off and land on airstrips of less than two kilometres, they're very versatile." The plane's pilot, Rickey Rau said Thor can fly fully loaded for at least five hours. "Depending on where the fire is, we can launch out of here fully loaded and do two to three runs on the fire before needing fuel," Mr Rau said. The plane will be based at the Richmond air base, but could also be moved to smaller bases at Williamtown, Tamworth, Dubbo and Canberra. The aircraft will be leased to NSW for two fire seasons as part of a $10 million State Government funding package. An ever bigger DC-10 air tanker will arrive in October. Commissioner Fitzsimmons said much of NSW was facing an "above normal" risk of bushfires this season. "That's largely caused by the moisture deficit," he said. He said there has been solid rainfall in recent months, but it was not likely to have a lasting effect. "With the onset of a strengthening El Nino still dominating the forecast for this season, the moisture is expected to be depleted fairly quickly, giving rise to a difficult fire season for 2015/16," he said.

SUMMARY:

According to ABC's Nick Dole, September 1st, the New South Wales Fire Service will use the first of two fire-fighting US-made air tankers this spring. Lack of moisture and El Nino signal more risk of fire. But the plane, reaching any fire in NSW quickly and dumping thousands of litres quickly, will make people feel safer. Fuel efficiency, range, speed, loading capacity and its ability to use short airstrips make it a singularly versatile fire-fighting tool.

YOUR TASK: We would like you to change this summary. To make it easier for you to make changes, we have pasted it in the box below so you can change it.

NOTE: The summary currently contains 76 words. The changed summary MUST NOT be between 70 and 94 words long.

[For human participants, text box with editable summary was provided]

[For GPT-4o] Text box:

According to ABC's Nick Dole, September 1st, the New South Wales Fire Service will use the first of two fire-fighting US-made air tankers this spring. Lack of moisture and El Nino signal more risk of fire. But the plane, reaching any fire in NSW quickly and dumping thousands of litres quickly, will make people feel safer. Fuel efficiency, range, speed, loading capacity and its ability to use short airstrips make it a singularly versatile fire-fighting tool.

Condition: Addition and Subtraction Equally Efficient + Valence of Instruction Positive

Here is a short news article about fire fighting in New South Wales, Australia. <u>Please read it carefully.</u>

[For human participants, this sentence was included: "**Note: The 'Next' button appears in 60 seconds.**"]

'Thor' aircraft, capable of dropping 15,000 litres of water, arrives in NSW ahead of bushfire season

(By Nick Dole, ABC News, Sep 1, 2015)

The New South Wales Rural Fire Service has unveiled its newest firefighting tool, a massive water-bombing aircraft nicknamed Thor, ahead of the bushfire season. The plane has just arrived from North America for the Australian bushfire season and performed a practise water-bombing exercise near Richmond air base in north-west Sydney. The air tanker can drop more than 15,000 litres of water or fire retardant on a blaze within a few seconds. It has a loaded cruising speed of 545 kilometres an hour, meaning it will be able to reach most fires in the state within one hour. Emergency Services Minister David Elliott said the aircraft would be a welcome sight for residents in fire-prone areas. "The people of NSW will be able to rest easy tonight, the first day of spring, knowing that Thor will be in the skies looking out for them," Mr Elliott said. RFS Commissioner Shane Fitzsimmons said the specially configured C-130 Hercules will dwarf the capability of the smaller water-bombing planes in the fleet. "This can carry five times the water of those machines," he said. "They can take off and land on airstrips of less than two kilometres, they're very versatile." The plane's pilot, Rickey Rau said Thor can fly fully loaded for at least five hours. "Depending on where the fire is, we can launch out of here fully loaded and do two to three runs on the fire before needing fuel," Mr Rau said. The plane will be based at the Richmond air base, but could also be moved to smaller bases at Williamtown, Tamworth, Dubbo and Canberra. The aircraft will be leased to NSW for two fire seasons as part of a $10 million State Government funding package. An ever bigger DC-10 air tanker will arrive in October. Commissioner Fitzsimmons said much of NSW was facing an "above normal" risk of bushfires this season. "That's largely caused by the moisture deficit," he said. He said there has been solid rainfall in recent months, but it was not likely to have a lasting effect. "With the onset of a strengthening El Nino still dominating

the forecast for this season, the moisture is expected to be depleted fairly quickly, giving rise to a difficult fire season for 2015/16," he said.

[Next page for human participants]

This is again the article you have just read. Below the article you will find a summary of the article.

'Thor' aircraft, capable of dropping 15,000 litres of water, arrives in NSW ahead of bushfire season

(By Nick Dole, ABC News, Sep 1, 2015)

The New South Wales Rural Fire Service has unveiled its newest firefighting tool, a massive water-bombing aircraft nicknamed Thor, ahead of the bushfire season. The plane has just arrived from North America for the Australian bushfire season and performed a practise water-bombing exercise near Richmond air base in north-west Sydney. The air tanker can drop more than 15,000 litres of water or fire retardant on a blaze within a few seconds. It has a loaded cruising speed of 545 kilometres an hour, meaning it will be able to reach most fires in the state within one hour. Emergency Services Minister David Elliott said the aircraft would be a welcome sight for residents in fire-prone areas. "The people of NSW will be able to rest easy tonight, the first day of spring, knowing that Thor will be in the skies looking out for them," Mr Elliott said. RFS Commissioner Shane Fitzsimmons said the specially configured C-130 Hercules will dwarf the capability of the smaller water-bombing planes in the fleet. "This can carry five times the water of those machines," he said. "They can take off and land on airstrips of less than two kilometres, they're very versatile." The plane's pilot, Rickey Rau said Thor can fly fully loaded for at least five hours. "Depending on where the fire is, we can launch out of here fully loaded and do two to three runs on the fire before needing fuel," Mr Rau said. The plane will be based at the Richmond air base, but could also be moved to smaller bases at Williamtown, Tamworth, Dubbo and Canberra. The aircraft will be leased to NSW for two fire seasons as part of a $10 million State Government funding package. An ever bigger DC-10 air tanker will arrive in October. Commissioner Fitzsimmons said much of NSW was facing an "above normal" risk of bushfires this season. "That's largely caused by the moisture deficit," he said. He said there has been solid rainfall in recent months, but it was not likely to have a lasting effect. "With the onset of a strengthening El Nino still dominating the forecast for this season, the moisture is expected to be depleted fairly quickly, giving rise to a difficult fire season for 2015/16," he said.

SUMMARY:

According to ABC's Nick Dole, September 1st, the New South Wales Fire Service will use the first of two fire-fighting US-made air tankers this spring. Lack of moisture and El Nino signal more risk of fire. But the plane, reaching any fire in NSW quickly and dumping thousands of litres quickly, will make people feel safer. Fuel efficiency, range, speed, loading capacity and its ability to use short airstrips make it a singularly versatile fire-fighting tool.

YOUR TASK: We would like you to <u>improve</u> this summary. To make it easier for you to make improvements, we have pasted it in the box below so you can improve it.

NOTE: The summary currently contains 76 words. The improved summary <u>MUST NOT</u> be between 64 and 88 words long.

[For human participants, text box with editable summary was provided]

[For GPT-4o] Text box:

According to ABC's Nick Dole, September 1st, the New South Wales Fire Service will use the first of two fire-fighting US-made air tankers this spring. Lack of moisture and El Nino signal more risk of fire. But the plane, reaching any fire in NSW quickly and dumping thousands of litres quickly, will make people feel safer. Fuel efficiency, range, speed, loading capacity and its ability to use short airstrips make it a singularly versatile fire-fighting tool.

Condition: Subtraction More Efficient + Valence of Instruction Positive

Here is a short news article about fire fighting in New South Wales, Australia. <u>Please read it carefully.</u>

[For human participants, this sentence was included: "**Note: The 'Next' button appears in 60 seconds.**"]

'Thor' aircraft, capable of dropping 15,000 litres of water, arrives in NSW ahead of bushfire season

(By Nick Dole, ABC News, Sep 1, 2015)

The New South Wales Rural Fire Service has unveiled its newest firefighting tool, a massive water-bombing aircraft nicknamed Thor, ahead of the bushfire season. The plane has just arrived from North America for the Australian bushfire season and performed a practise water-bombing exercise near Richmond air base in north-west Sydney. The air tanker can drop more than 15,000 litres of water or fire retardant on a blaze within a few seconds. It has a loaded cruising speed of 545 kilometres an hour, meaning it will be able to reach most fires in the state within one hour. Emergency Services Minister David Elliott said the aircraft would be a welcome sight for residents in fire-prone areas. "The people of NSW will be able to rest easy tonight, the first day of spring, knowing that Thor will be in the skies looking out for them," Mr Elliott said. RFS Commissioner Shane Fitzsimmons said the specially configured C-130 Hercules will dwarf the capability of the smaller water-bombing planes in the fleet. "This can carry five times the water of those machines," he said. "They can take off and land on airstrips of less than two kilometres, they're very versatile." The plane's pilot, Rickey Rau said Thor can fly fully loaded for at least five hours. "Depending on where the fire is, we can launch out of here fully loaded and do two to three runs on the fire before needing fuel," Mr Rau said. The plane will be based at the Richmond air base, but could also be moved to smaller bases at Williamtown, Tamworth, Dubbo and Canberra. The aircraft will be leased to NSW for two fire seasons as part of a $10 million State Government funding package. An ever bigger DC-10 air tanker will arrive in October. Commissioner Fitzsimmons said much of NSW was facing an "above normal" risk of bushfires this season. "That's largely caused by the moisture deficit," he said. He said there has been solid rainfall in recent months, but it was not likely to have a lasting effect. "With the onset of a strengthening El Nino still dominating

the forecast for this season, the moisture is expected to be depleted fairly quickly, giving rise to a difficult fire season for 2015/16," he said.

[Next page for human participants]

This is again the article you have just read. Below the article you will find a summary of the article.

'Thor' aircraft, capable of dropping 15,000 litres of water, arrives in NSW ahead of bushfire season

(By Nick Dole, ABC News, Sep 1, 2015)

The New South Wales Rural Fire Service has unveiled its newest firefighting tool, a massive water-bombing aircraft nicknamed Thor, ahead of the bushfire season. The plane has just arrived from North America for the Australian bushfire season and performed a practise water-bombing exercise near Richmond air base in north-west Sydney. The air tanker can drop more than 15,000 litres of water or fire retardant on a blaze within a few seconds. It has a loaded cruising speed of 545 kilometres an hour, meaning it will be able to reach most fires in the state within one hour. Emergency Services Minister David Elliott said the aircraft would be a welcome sight for residents in fire-prone areas. "The people of NSW will be able to rest easy tonight, the first day of spring, knowing that Thor will be in the skies looking out for them," Mr Elliott said. RFS Commissioner Shane Fitzsimmons said the specially configured C-130 Hercules will dwarf the capability of the smaller water-bombing planes in the fleet. "This can carry five times the water of those machines," he said. "They can take off and land on airstrips of less than two kilometres, they're very versatile." The plane's pilot, Rickey Rau said Thor can fly fully loaded for at least five hours. "Depending on where the fire is, we can launch out of here fully loaded and do two to three runs on the fire before needing fuel," Mr Rau said. The plane will be based at the Richmond air base, but could also be moved to smaller bases at Williamtown, Tamworth, Dubbo and Canberra. The aircraft will be leased to NSW for two fire seasons as part of a $10 million State Government funding package. An ever bigger DC-10 air tanker will arrive in October. Commissioner Fitzsimmons said much of NSW was facing an "above normal" risk of bushfires this season. "That's largely caused by the moisture deficit," he said. He said there has been solid rainfall in recent months, but it was not likely to have a lasting effect. "With the onset of a strengthening El Nino still dominating the forecast for this season, the moisture is expected to be depleted fairly quickly, giving rise to a difficult fire season for 2015/16," he said.

SUMMARY:

According to ABC's Nick Dole, September 1st, the New South Wales Fire Service will use the first of two fire-fighting US-made air tankers this spring. Lack of moisture and El Nino signal more risk of fire. But the plane, reaching any fire in NSW quickly and dumping thousands of litres quickly, will make people feel safer. Fuel efficiency, range, speed, loading capacity and its ability to use short airstrips make it a singularly versatile fire-fighting tool.

YOUR TASK: We would like you to improve this summary. To make it easier for you to make improvements, we have pasted it in the box below so you can improve it.

NOTE: The summary currently contains 76 words. The improved summary MUST NOT be between 70 and 94 words long.

[For human participants, text box with editable summary was provided]

[For GPT-4o] Text box:

According to ABC's Nick Dole, September 1st, the New South Wales Fire Service will use the first of two fire-fighting US-made air tankers this spring. Lack of moisture and El Nino signal more risk of fire. But the plane, reaching any fire in NSW quickly and dumping thousands of litres quickly, will make people feel safer. Fuel efficiency, range, speed, loading capacity and its ability to use short airstrips make it a singularly versatile fire-fighting tool.

SI 5: Estimates, odds ratios, confidence intervals, and p-values in Study 1

Experiment 1a

Table A7

Logistic Regression Results for the Interaction Agent x Solution Efficiency (Exp. 1a)

	Coefficients			Odds Ratio		*p*
	Estimate	SE	95 % CI	OR	95 % CI	
Intercept	0.17	0.22	[-0.26, 0.59]			.448
Agent						
human	0.48	0.34	[-0.18, 1.14]	1.62	[0.84, 3.17]	.151
Solution Efficiency						
sub more eff	0.65	0.32	[0.03, 1.28]	1.92	[1.03, 3.64]	.041
Agent * Solution Efficiency						
human * sub more eff	-2.19	0.49	[-3.17, -1.22]	0.11	[0.04, 0.29]	< .001

Note. Reference categories: Agent = GPT-4; Solution Efficiency = addition and subtraction equally efficient. OR = Odds Ratio; CI = Confidence Interval; sub more eff = subtraction more efficient (than addition).

Table A8

Logistic Regression Results for Solution Efficiency in Humans (Exp. 1a)

	Coefficients			Odds Ratio		*p*
	Estimate	SE	95 % CI	OR	95 % CI	
Intercept	0.66	0.06	[0.54, 0.77]			< .001
Solution Efficiency						
sub more eff	-0.36	0.08	[-0.52, -0.20]	0.69	[0.59, 0.82]	< .001

Note. Reference categories: Solution Efficiency = addition and subtraction equally efficient. OR = Odds Ratio; CI = Confidence Interval; sub more eff = subtraction more efficient (than addition).

Table A9

Logistic Regression Results for Solution Efficiency in GPT-4 (Exp. 1a)

	Coefficients			Odds Ratio		*p*
	Estimate	SE	95 % CI	OR	95 % CI	
Intercept	0.54	0.05	[0.44, 0.64]			< .001
Solution Efficiency						
sub more eff	0.15	0.07	[0.01, 0.30]	1.17	[1.01, 1.35]	.040

Note. Reference categories: Solution Efficiency = addition and subtraction equally efficient. OR = Odds Ratio; CI = Confidence Interval; sub more eff = subtraction more efficient (than addition).

Experiment 1b

Table A10

Logistic Regression Results for the Interaction Agent x Solution Efficiency (Exp. 1b)

	Coefficients			Odds Ratio		*p*
	Estimate	SE	95 % CI	OR	95 % CI	
Intercept	1.25	0.26	[0.73, 1.76]			<.001
Agent						
human	-0.94	0.34	[-1.61, -0.28]	0.39	[0.20, 0.75]	.005
Solution Efficiency						
sub more eff	1.02	0.45	[0.13, 1.91]	2.77	[1.17, 7.10]	.025
Agent * Solution Efficiency						
human * sub more eff	-1.76	0.55	[-2.83, -0.68]	0.17	[0.06, 0.50]	.001

Note. Reference categories: Agent = GPT-4; Solution Efficiency = addition and subtraction equally efficient. OR = Odds Ratio; CI = Confidence Interval; sub more eff = subtraction more efficient (than addition).

Table A11

Logistic Regression Results for Solution Efficiency in Humans (Exp. 1b)

	Coefficients			Odds Ratio		*p*
	Estimate	SE	95 % CI	OR	95 % CI	
Intercept	0.57	0.05	[0.47, 0.68]			< .001
Solution Efficiency						
sub more eff	-0.18	0.08	[-0.33, -0.03]	0.83	[0.72, 0.97]	.017

Note. Reference categories: Solution Efficiency = addition and subtraction equally efficient. OR = Odds Ratio; CI = Confidence Interval; sub more eff = subtraction more efficient (than addition).

Table A12

Logistic Regression Results for Solution Efficiency in GPT-4 (Exp. 1b)

	Coefficients			Odds Ratio		*p*
	Estimate	SE	95 % CI	OR	95 % CI	
Intercept	0.54	0.05	[0.44, 0.64]			< .001
Solution Efficiency						
sub more eff	0.15	0.07	[0.01, 0.30]	1.17	[1.01, 1.35]	.040

Note. Reference categories: Solution Efficiency = addition and subtraction equally efficient. OR = Odds Ratio; CI = Confidence Interval; sub more eff = subtraction more efficient (than addition).

Experiment 2a

Table A13

Logistic Regression Results for the Interaction Agent x Instruction Valence (Exp. 2a)

	Coefficients			Odds Ratio		*p*
	Estimate	SE	95 % CI	OR	95 % CI	
Intercept	-0.17	0.22	[-0.59, 0.26]			.448
Agent						
human	0.71	0.35	[0.04, 1.39]	2.04	[1.04, 4.05]	.039
Instruction Valence						
val positive	0.38	0.31	[-0.23, 0.98]	1.46	[0.80, 2.68]	.220
Agent * Instruction Valence						
human * val positive	-0.48	0.49	[-1.43, 0.48]	0.62	[0.24, 1.62]	.329

Note. Reference categories: Agent = GPT-4; Instruction Valence = valence of instruction neutral. OR = Odds Ratio; CI = Confidence Interval; val positive = valence of instruction positive.

Table A14

Logistic Regression Results for Instruction Valence in Humans (Exp. 2a)

	Coefficients			Odds Ratio		*p*
	Estimate	SE	95 % CI	OR	95 % CI	
Intercept	0.63	0.06	[0.51, 0.76]			< .001
Instruction Valence						
val positive	-0.02	0.09	[-0.20, 0.15]	0.98	[0.82, 1.16]	.797

Note. Reference categories: Instruction Valence = valence of instruction neutral. OR = Odds Ratio; CI = Confidence Interval; val positive = valence of instruction positive.

Table A15

Logistic Regression Results for Instruction Valence in GPT-4 (Exp. 2a)

	Coefficients			Odds Ratio		*p*
	Estimate	SE	95 % CI	OR	95 % CI	
Intercept	0.46	0.05	[0.35, 0.57]			< .001
Instruction Valence						
val positive	0.09	0.08	[-0.06, 0.24]	1.10	[0.95, 1.28]	.222

Note. Reference categories: Instruction Valence = valence of instruction neutral. OR = Odds Ratio; CI = Confidence Interval; val positive = valence of instruction positive.

Experiment 2b

Table A16

Logistic Regression Results for the Interaction Agent x Instruction Valence (Exp. 2b)

	Coefficients			Odds Ratio		*p*
	Estimate	SE	95 % CI	OR	95 % CI	
Intercept	1.18	0.26	[0.68, 1.68]			< .001
Agent						
human	-0.56	0.34	[-1.23, 0.12]	0.57	[0.29, 1.12]	.106
Instruction Valence						
val positive	2.13	0.64	[0.87, 3.39]	8.41	[2.73, 36.80]	< .001
Agent * Instruction Valence						
human * val positive	-1.73	0.73	[-3.15, -0.31]	0.18	[0.04, 0.66]	.017

Note. Reference categories: Agent = GPT-4; Instruction Valence = valence of instruction neutral. OR = Odds Ratio; CI = Confidence Interval; val positive = valence of instruction positive.

Table A17

Logistic Regression Results for Instruction Valence in Humans (Exp. 2b)

	Coefficients			Odds Ratio		*p*
	Estimate	SE	95 % CI	OR	95 % CI	
Intercept	0.65	0.05	[0.55, 0.75]			< .001
Instruction Valence						
val positive	0.08	0.07	[-0.06, 0.22]	1.09	[0.95, 1.25]	.242

Note. Reference categories: Instruction Valence = valence of instruction neutral. OR = Odds Ratio; CI = Confidence Interval; val positive = valence of instruction positive.

Table A18

Logistic Regression Results for Instruction Valence in GPT-4 (Exp. 2b)

	Coefficients			Odds Ratio		*p*
	Estimate	SE	95 % CI	OR	95 % CI	
Intercept	0.76	0.04	[0.69, 0.83]			< .001
Instruction Valence						
val positive	0.20	0.05	[0.10, 0.30]	1.22	[1.11, 1.35]	< .001

Note. Reference categories: Instruction Valence = valence of instruction neutral. OR = Odds Ratio; CI = Confidence Interval; val positive = valence of instruction positive.

SI 6: Estimates, odds ratios, confidence intervals, and p-values in Study 2

Experiment 3a

Table A19

Logistic Regression Results for the Interaction Agent x Solution Efficiency x Instruction Valence (Exp. 3a)

	Coefficients			Odds Ratio		*p*
	Estimate	SE	95 % CI	OR	95 % CI	
Intercept	-2.77	0.37	[-3.56, -2.12]			< .001
Agent						
human	1.86	0.46	[1.00, 2.80]	6.42	[2.72, 16.49]	< .001
Solution Efficiency						
sub more eff	-0.14	0.53	[-1.22, 0.91]	0.87	[0.30, 2.49]	.791
Instruction Valence						
val positive	-0.72	0.63	[-2.07, 0.46]	0.49	[0.13, 1.58]	.247
Agent * Solution Efficiency						
human * sub more eff	1.54	0.63	[0.30, 2.81]	4.67	[1.35, 16.59]	.015
Agent * Instruction Valence						
human * val positive	0.94	0.74	[-0.47, 2.47]	2.55	[0.62, 11.76]	.204
Solution Efficiency * Instruction Valence						
sub more eff * val positive	-0.15	0.94	[-2.07, 1.70]	0.86	[0.13, 5.45]	.870
Agent * Solution Efficiency * Instruction Valence						
human * sub more eff * val positive	-0.27	1.06	[-2.35, 1.87]	0.77	[0.10, 6.51]	.803

Note. Reference categories: Agent = GPT-4o; Solution Efficiency = addition and subtraction equally efficient; Instruction Valence = valence of instruction neutral. OR = Odds Ratio; CI = Confidence Interval; sub more eff = subtraction more efficient (than addition); val positive = valence of instruction positive.

Table A20

Logistic Regression Results for Solution Efficiency in Humans (Exp. 3a)

	Coefficients			Odds Ratio		*p*
	Estimate	SE	95 % CI	OR	95 % CI	
Intercept	-0.81	0.20	[-1.20, -0.43]			< .001
Solution Efficiency						
sub more eff	1.20	0.25	[0.72, 1.69]	3.31	[2.06, 5.40]	< .001

Note. Reference categories: Solution Efficiency = addition and subtraction equally efficient. OR = Odds Ratio; CI = Confidence Interval; sub more eff = subtraction more efficient (than addition).

Table A21

Logistic Regression Results for Solution Efficiency in GPT-4o (Exp. 3a)

	Coefficients			Odds Ratio		*p*
	Estimate	SE	95 % CI	OR	95 % CI	
Intercept	-3.07	0.30	[-3.70, -2.54]			< .001
Solution Efficiency						
sub more eff	-0.19	0.44	[-1.07, 0.67]	0.83	[0.34, 1.95]	.664

Note. Reference categories: Solution Efficiency = addition and subtraction equally efficient. OR = Odds Ratio; CI = Confidence Interval; sub more eff = subtraction more efficient (than addition).

Table A22

Logistic Regression Results for Instruction Valence in Humans (Exp. 3a)

	Coefficients			Odds Ratio		*p*
	Estimate	SE	95 % CI	OR	95 % CI	
Intercept	-0.06	0.16	[-0.37, 0.25]			.694
Instruction Valence						
val positive	-0.02	0.23	[-0.47, 0.43]	0.98	[0.63, 1.53]	.934

Note. Reference categories: Instruction Valence = valence of instruction neutral. OR = Odds Ratio; CI = Confidence Interval; val positive = valence of instruction positive.

Table A23

Logistic Regression Results for Instruction Valence in GPT-4o (Exp. 3a)

	Coefficients			Odds Ratio		*p*
	Estimate	SE	95 % CI	OR	95 % CI	
Intercept	-2.83	0.27	[-3.40, -2.35]			< .001
Instruction Valence						
val positive	-0.79	0.47	[-1.77, 0.09]	0.45	[0.17, 1.09]	.089

Note. Reference categories: Instruction Valence = valence of instruction neutral. OR = Odds Ratio; CI = Confidence Interval; val positive = valence of instruction positive.

Experiment 3b

Table A24

Logistic Regression Results for the Interaction Agent x Solution Efficiency x Instruction Valence (Exp. 3b)

	Coefficients			Odds Ratio		*p*
	Estimate	SE	95 % CI	OR	95 % CI	
Intercept	-4.20	0.71	[-6.00, -3.05]			< .001
Agent						
human	4.00	0.74	[2.78, 5.83]	54.63	[16.13, 341.72]	< .001
Solution Efficiency						
sub more eff	2.91	0.76	[0.91, 4.05]	8.94	[2.48,57.30]	.004
Instruction Valence						
val positive	-0.70	1.23	[-3.78, 1.66]	0.50	[0.02, 5.24]	.569
Agent * Solution Efficiency						
human * sub more eff	-1.67	0.81	[-3.59, -0.26]	0.19	[0.03, 0.77]	.039
Agent * Instruction Valence						
human * val positive	0.08	1.26	[-2.33, 3.20]	1.09	[0.10, 24.45]	.947
Solution Efficiency * Instruction Valence						
sub more eff * val positive	-15.86	561.38	-	0.00	-	.977
Agent * Solution Efficiency * Instruction Valence						
human * sub more eff * val positive	15.90	561.38	-	8,059,520	-	.977

Note. Reference categories: Agent = GPT-4o; Solution Efficiency = addition and subtraction equally efficient; Instruction Valence = valence of instruction neutral. Due to quasi-complete separation and numerical non-estimability, the confidence intervals for the Estimate and the OR of the interaction Solution Efficiency * Instruction Valence and the interaction Agent * Solution Efficiency * Instruction Valence are not interpretable and are thus not reported. OR = Odds Ratio; CI = Confidence Interval; sub more eff = subtraction more efficient (than addition); val positive = valence of instruction positive.

Table A25

Logistic Regression Results for Solution Efficiency in Humans (Exp. 3b)

	Coefficients			Odds Ratio		*p*
	Estimate	SE	95 % CI	OR	95 % CI	
Intercept	-0.51	0.14	[-0.79, -0.24]			< .001
Solution Efficiency						
sub more eff	0.53	0.19	[0.15, 0.91]	1.70	[1.16, 2.48]	.006

Note. Reference categories: Solution Efficiency = addition and subtraction equally efficient. OR = Odds Ratio; CI = Confidence Interval; sub more eff = subtraction more efficient (than addition).

Table A26

Logistic Regression Results for Solution Efficiency in GPT-4o (Exp. 3b)

	Coefficients			Odds Ratio		p
	Estimate	SE	95 % CI	OR	95 % CI	
Intercept	-4.49	0.58	[-5.88, -3.53]			< .001
Solution Efficiency						
sub more eff	1.72	0.64	[0.61, 3.19]	5.61	[1.84, 24.31]	.007

Note. Reference categories: Solution Efficiency = addition and subtraction equally efficient. OR = Odds Ratio; CI = Confidence Interval; sub more eff = subtraction more efficient (than addition).

Table A27

Logistic Regression Results for Instruction Valence in Humans (Exp. 3b)

	Coefficients			Odds Ratio		p
	Estimate	SE	95 % CI	OR	95 % CI	
Intercept	0.07	0.14	[-0.20, 0.34]			.628
Instruction Valence						
val positive	-0.58	0.19	[-0.97, -0.21]	0.56	[0.38, 0.81]	.003

Note. Reference categories: Instruction Valence = valence of instruction neutral. OR = Odds Ratio; CI = Confidence Interval; val positive = valence of instruction positive.

Table A28

Logistic Regression Results for Instruction Valence in GPT-4o (Exp. 3b)

	Coefficients			Odds Ratio		p
	Estimate	SE	95 % CI	OR	95 % CI	
Intercept	-2.64	0.24	[-3.15, -2.19]			< .001
Instruction Valence						
val positive	-2.96	1.03	[-5.85, -1.37]	0.05	[0.003, 0.25]	.004

Note. Reference categories: Instruction Valence = valence of instruction neutral. OR = Odds Ratio; CI = Confidence Interval; val positive = valence of instruction positive.

Experiment 3a (only accurate solutions)

Table A29

Logistic Regression Results for the Interaction Agent x Solution Efficiency x Instruction Valence (Exp. 3a, only accurate solutions)

	Coefficients			Odds Ratio		*p*
	Estimate	SE	95 % CI	OR	95 % CI	
Intercept	-2.73	0.42	[-3.67, -1.99]			< .001
Agent						
human	1.78	0.51	[0.83, 2.86]	5.92	[2.29, 17.38]	< .001
Solution Efficiency						
sub more eff	-0.27	0.66	[-1.66, 1.02]	0.77	[0.19, 2.78]	.689
Instruction Valence						
val positive	-0.97	0.83	[-2.91, 0.53]	0.38	[0.05, 1.70]	.242
Agent * Solution Efficiency						
human * sub more eff	2.47	0.78	[0.97, 4.06]	11.82	[2.63, 58.10]	.001
Agent * Instruction Valence						
human * val positive	1.31	0.92	[-0.39, 3.38]	3.71	[0.68, 29.40]	.156
Solution Efficiency * Instruction Valence						
sub more eff * val positive	1.15	1.08	[-0.89, 3.47]	3.14	[0.41, 32.09]	.288
Agent * Solution Efficiency * Instruction Valence						
human * sub more eff * val positive	-2.13	1.21	[-4.68, 0.18]	0.12	[0.01, 1.19]	.079

Note. Reference categories: Agent = GPT-4o; Solution Efficiency = addition and subtraction equally efficient; Instruction Valence = valence of instruction neutral. OR = Odds Ratio; CI = Confidence Interval; sub more eff = subtraction more efficient (than addition); val positive = valence of instruction positive.

Table A30

Logistic Regression Results for Solution Efficiency in Humans (Exp. 3a, only accurate solutions)

	Coefficients			Odds Ratio		*p*
	Estimate	SE	95 % CI	OR	95 % CI	
Intercept	-0.79	0.20	[-1.19, -0.40]			< .001
Solution Efficiency						
sub more eff	1.70	0.27	[1.17, 2.25]	5.49	[3.23, 9.50]	< .001

Note. Reference categories: Solution Efficiency = addition and subtraction equally efficient. OR = Odds Ratio; CI = Confidence Interval; sub more eff = subtraction more efficient (than addition).

Table A31

Logistic Regression Results for Solution Efficiency in GPT-4o (Exp. 3a, only accurate solutions)

	Coefficients			Odds Ratio		*p*
	Estimate	SE	95 % CI	OR	95 % CI	
Intercept	-3.07	0.36	[-3.87, -2.43]			< .001
Solution Efficiency						
sub more eff	0.17	0.50	[-0.81, 1.17]	1.19	[0.44, 3.23]	.731

Note. Reference categories: Solution Efficiency = addition and subtraction equally efficient. OR = Odds Ratio; CI = Confidence Interval; sub more eff = subtraction more efficient (than addition).

Table A32

Logistic Regression Results for Instruction Valence in Humans (Exp. 3a, only accurate solutions)

	Coefficients			Odds Ratio		*p*
	Estimate	SE	95 % CI	OR	95 % CI	
Intercept	0.20	0.17	[-0.14, 0.54]			.260
Instruction Valence						
val positive	-0.13	0.25	[-0.62, 0.36]	0.88	[0.54, 1.44]	.604

Note. Reference categories: Instruction Valence = valence of instruction neutral. OR = Odds Ratio; CI = Confidence Interval; val positive = valence of instruction positive.

Table A33

Logistic Regression Results for Instruction Valence in GPT-4o (Exp. 3a, only accurate solutions)

	Coefficients			Odds Ratio		*p*
	Estimate	SE	95 % CI	OR	95 % CI	
Intercept	-2.84	0.33	[-3.55, -2.26]			< .001
Instruction Valence						
val positive	-0.32	0.50	[-1.35, 0.67]	0.73	[0.26, 1.94]	.532

Note. Reference categories: Instruction Valence = valence of instruction neutral. OR = Odds Ratio; CI = Confidence Interval; val positive = valence of instruction positive.

SI 7 Additional Analyses Based on Accurate Solutions

Table A34

Logistic Regression Analysis Results for Experiment 3a (Accurate Solutions only)

Effect	*N*	$\chi^2(1)$	*p*
Solution Efficiency	849	35.47	< .001
Instruction Valence		0.82	.364
Agent		199.75	< .001
Solution Efficiency x Instruction Valence		1.11	.292
Solution Efficiency x Agent		7.07	.008
Instruction Valence x Agent		0.06	.802
Solution Efficiency x Instruction Valence x Agent		3.27	.070

Note. For GPT-4o, analyses were based on the grid-based response format.
